# TiO_2_ Gas Sensors Combining Experimental and DFT Calculations: A Review

**DOI:** 10.3390/nano12203611

**Published:** 2022-10-14

**Authors:** Zirui Yan, Yaofang Zhang, Weimin Kang, Nanping Deng, Yingwen Pan, Wei Sun, Jian Ni, Xiaoying Kang

**Affiliations:** 1 State Key Laboratory of Separation Membranes and Membrane Processes, Tiangong University, Tianjin 300387, China; 2 School of Physical Science and Technology, Tiangong University, Tianjin 300387, China; 3 School of Textile Science and Engineering, Tiangong University, Tianjin 300387, China; 4 Department of Electronic Science and Technology, College of Electronic Information and Optical Engineering, Nankai University, Tianjin 300350, China

**Keywords:** TiO_2_, gas sensors, DFT, ab initio

## Abstract

Gas sensors play an irreplaceable role in industry and life. Different types of gas sensors, including metal-oxide sensors, are developed for different scenarios. Titanium dioxide is widely used in dyes, photocatalysis, and other fields by virtue of its nontoxic and nonhazardous properties, and excellent performance. Additionally, researchers are continuously exploring applications in other fields, such as gas sensors and batteries. The preparation methods include deposition, magnetron sputtering, and electrostatic spinning. As researchers continue to study sensors with the help of modern computers, microcosm simulations have been implemented, opening up new possibilities for research. The combination of simulation and calculation will help us to better grasp the reaction mechanisms, improve the design of gas sensor materials, and better respond to different gas environments. In this paper, the experimental and computational aspects of ${\mathrm{TiO}}_{2}$ are reviewed, and the future research directions are described.

## 1. Introduction

**Figure 1:**
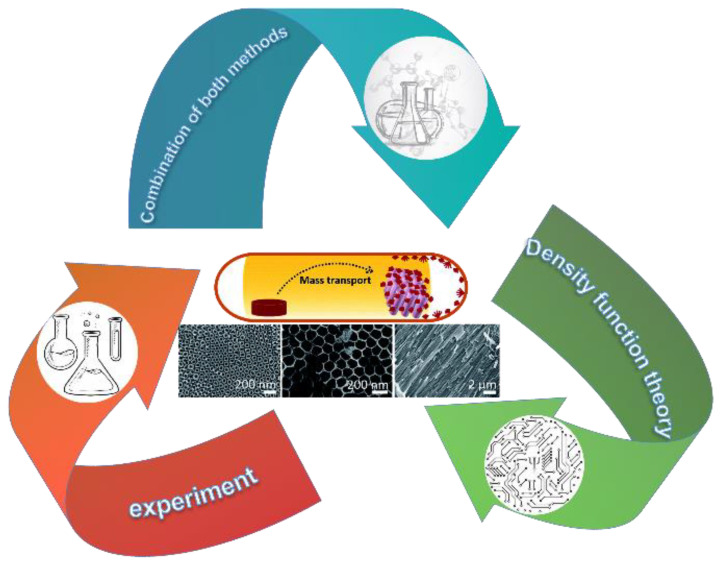
The relationship between the way gas sensors is studied based on the views reviewed in this paper. Schematic diagram of gas-sensitive reaction process and SEM image of ${\mathrm{TiO}}_{2}$ nanotube material. Reprinted with permission from Ref. [11].

Humans cannot live without gas. However, some of these gases are toxic and harmful even though they may be colorless and odorless. When these gases are present in our environment, it is essential to detect them immediately to protect us from their hazards. Research on gas sensors has long been ongoing and of increasing interest due to their role in a wide range of fields, including laboratory health and safety, gas detection, observation, and environmental investigation [1], with even lithium-ion batteries [2] or solid-oxide fuel cells [3] using them. The current mainstream gas sensors available today can be classified as metal-oxide sensors (MOS) [4], polymer sensors, carbon nanotube sensors, and spectral analysis sensors [5]. Among these sensing materials, metal-oxide (MOX) semiconductors (${\mathrm{TiO}}_{2}$, ${\mathrm{SnO}}_{2}$, ZnO, etc.) are the most commonly used because of their low cost of preparation and high sensitivity to different gases [6]. Even though ${\mathrm{SnO}}_{2}$ gas sensors have been widely studied and are more mature, numerous studies still show that ${\mathrm{TiO}}_{2}$, which is also a broadband semiconductor, has a better safety profile than conventional ${\mathrm{SnO}}_{2}$ gas sensors [7]. The response of ${\mathrm{TiO}}_{2}$ gas sensors to gases may be much better than that of ${\mathrm{SnO}}_{2}$ gas sensors due to lower Schottky defects in the same environment [8]. Furthermore, if one can take advantage of the unique light-sensitive, photocatalytic, and spin-magnetic properties of ${\mathrm{TiO}}_{2}$, or combine the spin magnetism of ${\mathrm{TiO}}_{2}$ [9] with the magnetism of the gas [10], the response value of ${\mathrm{TiO}}_{2}$ gas sensors may be several times better than that of ${\mathrm{SnO}}_{2}$ gas sensors; thus, this concept needs a combination of theoretical guidance and experimental verification as envisioned in Figure 1 [11]. The major structure of a gas sensor consists of a highly conductive metal electrode on a sensing substrate and a sensor made of sensing material [6]. Intriguing and noteworthy is the fact that the connection effect between the sensing substrate electrodes and the material may affect the gas sensor performance. The stronger the connection between the electrode and the material, the more the gas sensor performance may dramatically improve [12]. Although there are some limitations in the measurement range of metal-oxide semiconductor sensors, some of them are well accepted and used because of their advantages of nontoxicity, low costs, and high stability in harsh environments [13].

**Figure 2:**
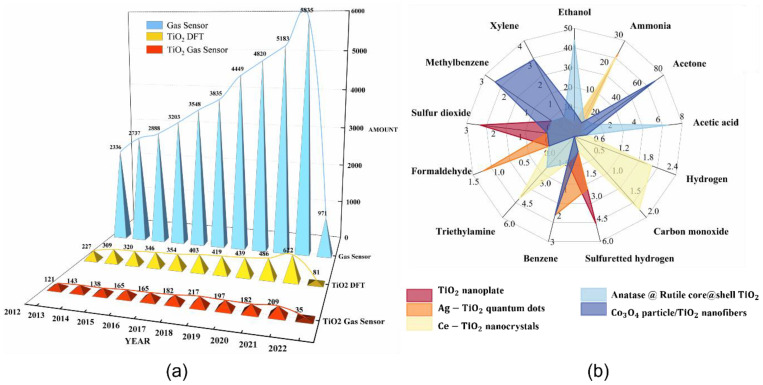
(**a**) The trend of the number of SCI articles retrieved in recent years; blue indicates the search results with the gas sensor as the keyword, the yellow part is the search results with ${\mathrm{TiO}}_{2}$ DFT as the keyword, and the red part is the search results with ${\mathrm{TiO}}_{2}$ gas sensor as the keyword. (**b**) Selectivity of ${\mathrm{TiO}}_{2}$ doped with different materials for sensing gases.

${\mathrm{TiO}}_{2}$ has been used as an industrial catalyst due to its high catalytic activity and selectivity [14]. The photocatalytic performance of ${\mathrm{TiO}}_{2}$ gives more satisfactory results under UV irradiation compared to other oxides, allowing for a better reaction of $O_{2}^{-}$ with bowls of paraffin, olefins, CO, ${\mathrm{SO}}_{2}$, and NO [15]. The quantum size effect and surface effect make ${\mathrm{TiO}}_{2}$ exhibit more excellent physical and chemical properties when ${\mathrm{TiO}}_{2}$ reaches the nanoscale, giving it unique electrical, optical, catalytic [16], and gas-sensitive properties [17] and dramatically enhancing the performance, which enables more extensive and in-depth studies. ${\mathrm{TiO}}_{2}$ holds much more potential for researchers to explore [18], and as they have never slackened their experimental and theoretical research on Ti, as depicted in Figure 2a, there continues to be a sustained growth in the number of articles related to ${\mathrm{TiO}}_{2}$ as well as gas sensors. Moreover, compared with ordinary ${\mathrm{SnO}}_{2}$ gas sensors, ${\mathrm{TiO}}_{2}$ gas sensors are less affected by humidity [19]. It is these features that make it possible to use ${\mathrm{TiO}}_{2}$ for gas sensors and insulation testing [20]. In the study of gas sensors, many researchers have tried a variety of methods [21]. Sensitivity, response time, and gas-sensing performance all improved [22]. In particular, for toxic gases that are harmful to human health, gas sensors must be used for detection [23]. In Table 1, the authors summarize the results of gas-sensitivity response and operating temperature, and response/recovery time for different preparation methods or material doping. As some of the data in Table 1 show, ${\mathrm{TiO}}_{2}$ gas sensors respond well to most gases, including toxic gases. The need to detect atmospheric or environmental gases in most cases of practical use requires the gas sensor to be selective, which can severely limit the application of the device if it does not have the anti-interference performance against the detected unknown gases. As shown in Figure 2b, the authors enumerate the selectivity of five different materials (red is ${\mathrm{TiO}}_{2}$ particles [24], orange is Ag-doped ${\mathrm{TiO}}_{2}$ quantum dots [25], light yellow is Ce-doped ${\mathrm{TiO}}_{2}$ nanocrystals [26], light blue is ${\mathrm{TiO}}_{2}$ nanoshells with anatase and rutile phases [27], and dark blue is ${\mathrm{Co}}_{3}O_{4}$ particles loaded on ${\mathrm{TiO}}_{2}$ fibers [28]) that were prepared by combining them with ${\mathrm{TiO}}_{2}$ for various gases, all of which are common but potentially more hazardous gases. As can be seen, each material responds to more than one gas to varying degrees, which can also be seen as a difference in the sensitivity of the gas-sensitive materials to different gases. Although these reactions are explained chemically in detail, the explanation at the more microscopic physical level is not very adequate, and therefore, physical-theoretical studies on ${\mathrm{TiO}}_{2}$ gas-sensitive detection are being conducted and deepened simultaneously with experiments [29]. Since 1998, it has been reported that ${\mathrm{TiO}}_{2}$ acts as a gas-sensing material [30]. As the research progressed, various types of materials were combined with ${\mathrm{TiO}}_{2}$ to achieve a better gas-sensing response, and the study by Zhenyu Li et al. [31] showed that it already responded to gas at room temperature. Meanwhile, in the wake of the sophistication of research methods and the accumulation of research results, the related research is expanding and deepening, and many excellent research results related to this topic were already summarized in [32]. It was only a few years ago that the DFT became well integrated into experiments to help researchers develop their studies more rapidly. Therefore, this paper combines the results of DFT theoretical calculations and mainly reviews the research on ${\mathrm{TiO}}_{2}$ gas sensors in recent years, with ${\mathrm{TiO}}_{2}$ gas sensors preceding this paper.

**Figure 3:**
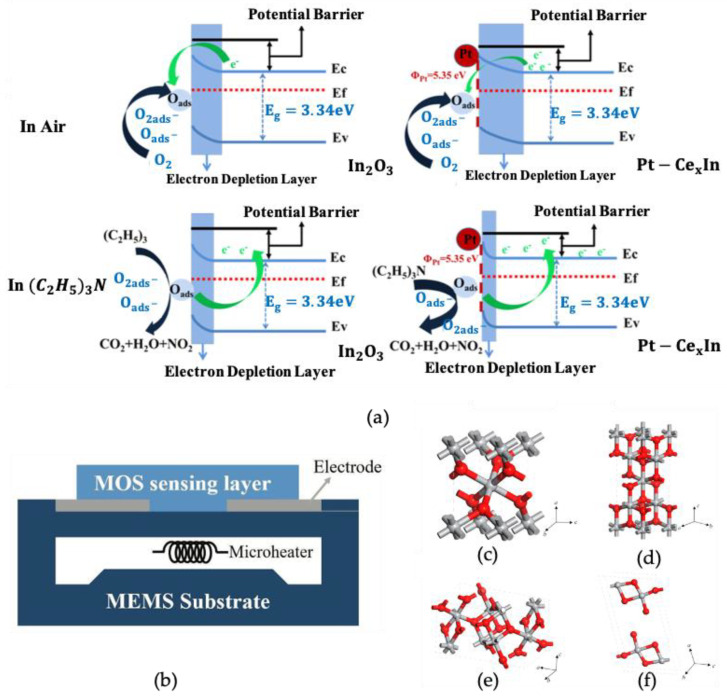
(**a**) Illustration of semiconductor sensor action mechanism. Reprinted with permission from Ref. [45]. Copyright 2020, American Chemical Society. (**b**) Structure diagram of gas sensor, microelectromechanical system (MEMS) is integrated into sensors. Reprinted with permission from Ref. [48]. Crystal structures of ${\mathrm{TiO}}_{2}$: (**c**) rutile; (**d**) anatase; (**e**) brookite; (**f**) ${\mathrm{TiO}}_{2}$(B)(monoclinic); red spheres represent O atoms, grey spheres represent Ti atoms. Reprinted with permission from Ref. [50].

The MOS gas sensor is designed based on the change in conductivity of the sensor material under the action of reducing or oxidizing gas (Figure 3a [45]). As the temperature increases, the conductivity of the sensor also increases. However, this temperature variability is not linear, and once the temperature threshold is exceeded, the gas-sensitive properties of the material on the sensor drop sharply, probably due to the temperature-dependent effect of the kinetic energy required for gas–surface interactions [46]. Likewise, this effect can be caused by changes in ambient temperature on the surface of the gas sensor, but either way, it can affect the gas sensor [47]. Each material has its optimum temperature range, called the operating temperature. At this temperature range, the performance of the sensor is considered to be optimal [48]. The overall structure of the gas sensor is shown in Figure 3b [48]. It is mainly composed of gas-sensitive elements, the electrode of the sensing substrate, heating elements, and other corresponding components [45]. One of the more widely accepted explanations is the adsorbed oxygen model, where the sensitivity of metal-oxide-based sensors depends mainly on the available adsorption sites and the amount of oxygen adsorbed [49]; this is concerned with the action of the $O_{2\left(\mathrm{ads}\right)}^{-}$ and $O_{\left(\mathrm{ads}\right)\;}^{-}$ ions on the surface of the material, that is, the metal-oxide surface material first reacts with oxygen molecules or water molecules in the air and uses the reacted $O_{2\left(\mathrm{ads}\right)}^{-}$ and $O_{\left(\mathrm{ads}\right)}^{-}$ ions to then react with other gases [49], as demonstrated in Equations (1)–(4) [43]:

(1) $$O_{2\left(\mathrm{gas}\right)}\to O_{2\left(\mathrm{ads}\right)}$$

(2) $$O_{2\left(\mathrm{ads}\right)}+e^{-}\to O_{2\left(\mathrm{ads}\right)}^{-}\;\left(T_{\mathrm{opt}.}<100\;{}^{\circ}C\right)$$

(3) $$O_{2\left(\mathrm{ads}\right)}^{-}+e^{-}\to 2O_{\left(\mathrm{ads}\right)}^{-}\;\left(100\;{}^{\circ}C<T_{\mathrm{opt}.}<300\;{}^{\circ}C\right)$$

(4) $$O_{\left(\mathrm{ads}\right)}^{-}+e^{-}\to O_{\left(\mathrm{ads}\right)}^{2-}\;\left(T_{\mathrm{opt}.}>300\;{}^{\circ}C\right)$$

The important parameters for judging the performance of gas-sensitive sensors include response value and response recovery time; the response value (S) for an n-type semiconductor can be expressed by Equation (5) [47], in which $R_{\mathrm{air}}$ is the resistance value measured by the device in the air environment and $R_{\mathrm{gas}}$ is the resistance value measured by the device in the monitored gas environment, and the response/recovery time can be calculated using Equation (6) [51]:

(5) $$S=\frac{R_{\mathrm{air}}}{R_{\mathrm{gas}}}$$

(6) tresponserecovery=t90% of total−tinitial

In Equation (6), $t_{\mathrm{response}}$ is the response time, which represents the time it takes for the device to reach 90% of its complete response in the air from the time it is exposed to the detection gas; $t_{\mathrm{recovery}}$ is the recovery time, which is similar to the response time in that it represents the time it takes for the device’s resistance value to return to 90% of its initial state in the detection gas environment; and they can both be expressed by $t_{90\%\;\mathrm{of}\;\mathrm{total}}-t_{\mathrm{initial}}.$ The authors have listed several common gases in Table 1 to visualize and compare the response sensitivity of the gas sensors. The preparation method, operating temperature, and response/recovery time are contained in Table 1. It is generally desirable to achieve lower operating temperatures, higher response sensitivity, and shorter response/recovery times for gas sensors, but it is admittedly tremendously challenging to improve the results of these critical factors simultaneously. Therefore, we need more analytical approaches such as the first principle to find the root cause of the obstacles to research progress.

Computer technology is also developing rapidly at a time when the level of experimentation is constantly improving. It can be said that a computer’s help is indispensable in every aspect now, including the experimental part. In the same way, the theory of physics has made astonishing progress. The development of quantum mechanics has promoted the rise and application of the density functional theory (DFT). After deducing the famous Kohn–Sham (K-S) equation by local density approximation (LDA), the first-principle calculation based on DFT has become a powerful material development tool [52]. Using computer technology, the first-principle calculation is not only the essential explanation of the existing experimental results, but it can also be used to predict the early stages of an experiment or to simulate the whole process to analyze the results more accurately. Xiaoyu Tang et al. [53] investigated the results of doping calculations for three elements, namely, Re, Ce, and Gd, using first-principle DFT calculations. The results of the doping calculations for Re showed that the doping of this element can effectively reduce the forbidden bandwidth and redshift the absorption spectrum; the doping of Ce introduces impurity energy levels near the valence band, thus forming a recombination center for photogenerated electron and hole pairs at this position; and the doping of Gd creates the ${\mathrm{TiO}}_{2}$ system with a significant nonlocal built-in electric field, which is more favorable to change the charge separation and optical catalytic activity. The authors then conducted experiments to verify the results and found that the results obtained were consistent with the theoretical predictions when 5 wt% Gd-doped ${\mathrm{TiO}}_{2}$ could degrade more than 90% of methylene blue (MB) within 10 min under the light condition of a 405 nm wavelength, and the characterization showed that gadolinium oxide was distributed on the surface of ${\mathrm{TiO}}_{2}$, which enhanced the surface-charge transfer process and improved the catalytic effect. Combining first-principle calculations with experiments seems to be a good choice.

There are several experimental methods to improve the performance of ${\mathrm{TiO}}_{2}$ gas sensors. With the application of computer technology, the results are obtained from different angles. ${\mathrm{TiO}}_{2}$ is an n-type semiconductor [6], which exists in three polymorphisms, the rutile phase (tetragonal, P42/mnm), anatase phase (tetragonal, I41/amd) [54], and brookite phase (orthorhombic, pbca) [55], and the lattice constants differ between the different phases [50]; these structures are illustrated in Figure 3c–f [50]. In addition, the transformation of ${\mathrm{TiO}}_{2}$ from the anatase phase to the rutile phase starts to occur when the temperature reaches 600 °C [19]. The bandwidth of the three crystals is 3.02 eV, 3.2 eV, and 2.96 eV [50]. However, such a large bandgap requires more energy to excite the electron from the valence to conduction, which is not conducive to the rapid and efficient operation of ${\mathrm{TiO}}_{2}$ gas sensors. Therefore, modifications required to reduce the ${\mathrm{TiO}}_{2}$ bandgap or to attempt to improve the specific surface area of the sensor are also effective ways to make the application more efficient [56]. There are two kinds of strategies to improve the sensing performance of the ${\mathrm{TiO}}_{2}$ gas sensor. The first is to increase the specific surface area. The second is to narrow the bandgap [40]. It is found that there are significant differences in modification methods, including a simple physical structure modification and doping modification.

**Figure 4:**
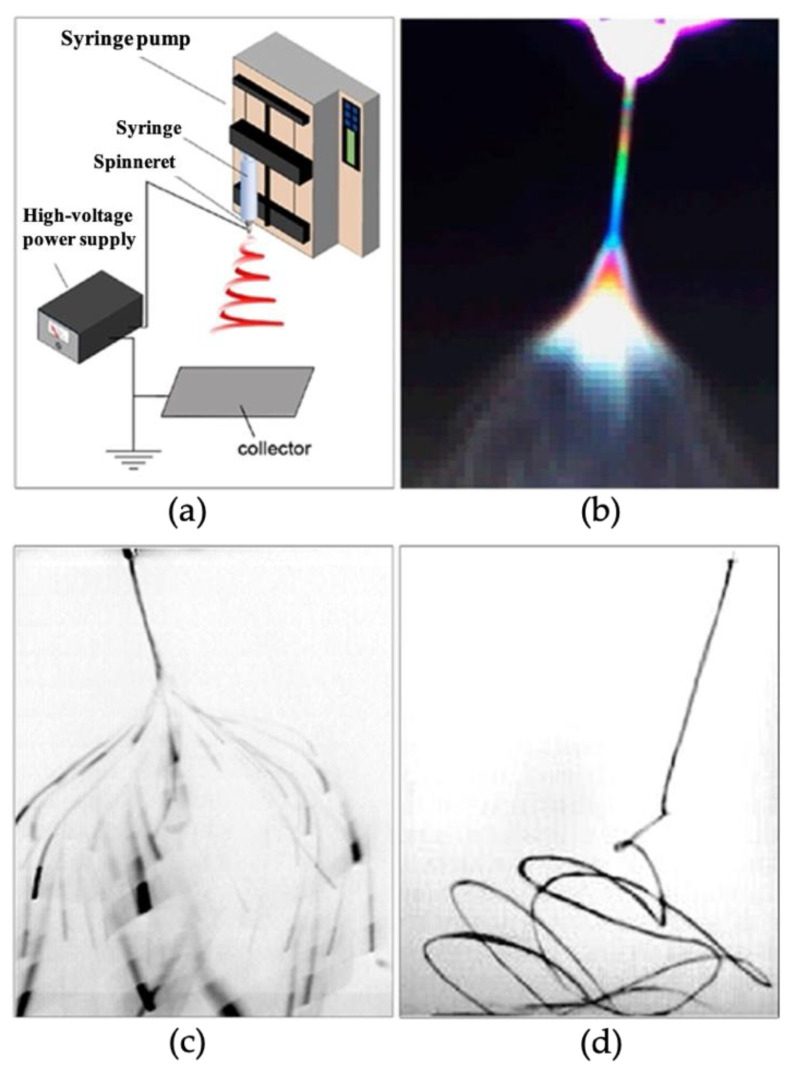
Schematic diagram of the electrospinning process. (**a**) schematic diagram of the electrospinning device, (**b**) electrospinning diagram under interference color photography technique, (**c**) electrospinning camera diagram with an exposure time of 33 ms, (**d**) high-speed camera electrostatic spinning jet diagram with an exposure time of 0.1 ms. Reprinted with permission from Ref. [59]. Copyright 2017, American Chemical Society.

The performance of the sensor can be improved by comparing the experimental results, as different experimental methods can obtain different results. In the past, in the manufacturing of gas sensors, the sensing material was uniformly covered on the substrate because the electrical conductivity of metal oxides varies with the adsorption and desorption of gas molecules, but it turns out that this may not be good enough [57]. Common methods are CVD and ALD methods, solid-phase reactions, electrochemical deposition, chemical sedimentation, hydrothermal/solvent thermal techniques, sol–gel, electrospinning [50], and flame aerosol processes [58]. As shown in Figure 4a–d [59], the electrostatic spinning method stretches the precursor solution into thin filamentary fibers through the action of a strong electric field, and the diameter of such filamentary fibers usually varies from a few tens of nanometers to a few micrometers.

In general, the excellent advantages of ${\mathrm{TiO}}_{2}$ in terms of nontoxicity, low cost, and high stability in harsh environments have attracted researchers to study it in more depth, not only in terms of experimentally unfolding research, but also in terms of theoretical studies, such as those analyzing the academic potential of ${\mathrm{TiO}}_{2}$ and its application potential in gas sensing and photocatalysis. Undeniably, this basic research has progressed over a long time and has achieved many excellent results, which has laid a solid foundation for future research; however, it also exposes the problems that still exist and the areas for improvement, explaining our need for more in-depth theoretical help to guide us in conducting analyses, which is why more and more studies include calculations such as DFT. The increasing proportion of theoretical calculations in research requires researchers to develop a new understanding of the integrated experimental and theoretical approaches.

## 2. TiO_2_ Nanostructure-Based Gas Sensors

### 2.1. Pure $TiO_{2}$ Gas Sensors

**Figure 5:**
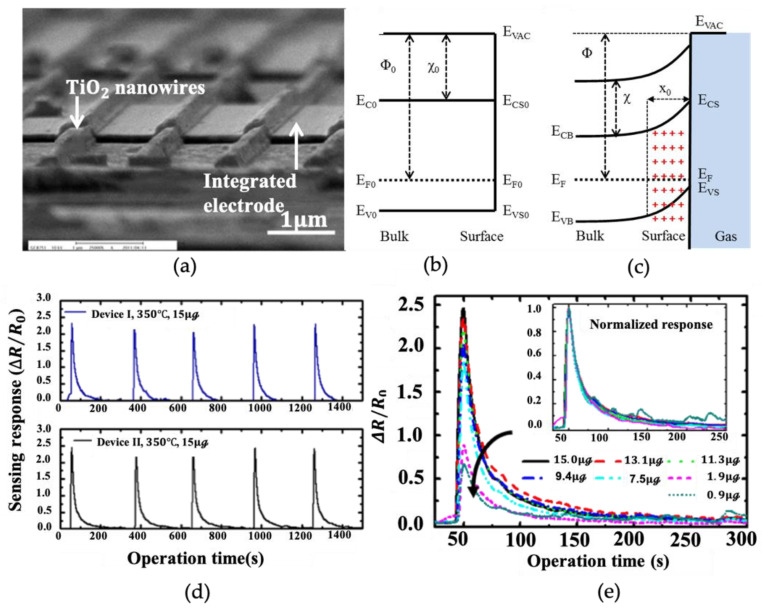
(**a**) ${\mathrm{TiO}}_{2}$ nanowire on the sensor surface. The picture is taken by SEM, showing the formation of a space-charge region of p-type ${\mathrm{TiO}}_{2}$ energy-band model before adsorption; (**b**) after adsorption; (**c**) ambient oxygen; (**d**) reliability testing of sensors; (**e**) the sensor transient response of ${\mathrm{TiO}}_{2}\;$ nanowire. Reprinted with permission from Ref. [56].

Various materials have been obtained through different methods [60], including the study of low-dimensional materials that affect the properties of materials [52]. Weicheng Tian et al. [56] increased the surface-to-volume ratio of the sensor and increased the exposure area of the gas-sensing material in the gas by changing the shape of its structure instead of the doping gas (Figure 5a). To achieve the same high-precision control as the chemical growth method, Tian studied the growth of nanowire between the Cr/Au electrode-film deposition on the sensing substrate and lithography on the surface of the ${\mathrm{TiO}}_{2}$ sensor. The analysis of the energy-band structure of this architecture in various environmental conditions (Figure 5b,c) is given by Tian. To improve the sensitivity, a 1-h annealing process was carried out at 450 °C; the optimum working temperature is 300 °C. Tian’s important parameter is response time, which is expressed as rising/recovery time $\left(\Delta R/R_{0}\right)$. In this article, the sensitivity to ethanol is 3.2/17.5 s, which is almost 2.5 times that of ordinary sensor times. On this basis, the sensor has a considerable lifetime, as shown in Figure 5d, and there are few signs of deterioration after several tests. This result proves, unquestionably, that gas sensors have a long service life. Response times for different concentrations of ethanol gas at a fixed temperature are extremely close to this structure and do not suffer as in other construction sensors (Figure 5e).

In addition, unadulterated nanoparticles are used to help improve the performance of gas sensing. Azhar Ali Haidry et al. [61] used magnetron sputtering; first, they sputtered out Pt beneficiation electrodes on the sensing sapphire substrate, then, grew ${\mathrm{TiO}}_{2}$ using the high-purity Ti target material in an unbalanced DC magnetically induced environment. The sensitivity of gas sensors is improved by the non-in situ annealing of tubular furnaces in 600 °C or 900 °C air. Comparing the results of annealing at different temperatures, it is found that the films have a preferential orientation relative to the substrate. At the same time, we can see intuitively from Azhar Ali Haidry’s work [61] that particles increase with the increase in temperature. The results of the hydrogen sensitivity test also show that when annealed at 900 °C, it has a high sensitivity at a working temperature below  1500 ppm. Wei Guo et al. [24] prepared ${\mathrm{TiO}}_{2}$-sensing materials with highly reactive nanoplate structures and investigated and improved the gas-sensitive properties of a series of toxic gases. There are also flame aerosol processes that can improve the response; when using the flame fabrication method, the size, crystallinity, and morphology of ${\mathrm{TiO}}_{2}$ can be controlled by varying the high-temperature residence time of the particles in the flame [19]. During the time when nanoparticles are made, Erik Tolmachoff et al. [62] used this method to prepare nanoporous ${\mathrm{TiO}}_{2}$ thin-film gas-sensing materials to produce ${\mathrm{TiO}}_{2}$ particles with a diameter of about 9 nm, which improves the response at an order of magnitude compared to commercial sensing films made of ${\mathrm{TiO}}_{2}$ particles with a diameter of 25 nm. The main reason is that this method allows for the preparation of smaller-diameter particles to obtain a larger specific surface area, which is a key factor in improving the gas-sensing effect.

Electrostatic spinning is one of the most commonly used methods to produce nanofilaments. Electrostatic spinning technology has also been used in many fields, including in the study of gas-sensitive materials, by being combining with other technologies. Fibers that are 20 nm nanometers in diameter have been made by Jin-Ah Park [63]. The mixed ${\mathrm{TiO}}_{2}/\mathrm{PVP}$ fiber was obtained by electrostatic spinning technology. The fibers obtained by electrospinning are between 350 nm and 500 nm in diameter and have irregular meshes. After preparing the nanofibers, different calcination temperatures were studied, and the gradients and degrees of three different temperatures were set as 400 °C, 600 °C, and 800 °C. In the process of preparing nanofibers by electrostatic spinning, the purpose of roasting is not only to heat PVP in the mixed fibers, but also to make them burn. As a result, when the fibers are calcined at different temperatures, the surface of the fibers becomes rougher as the calcination temperature increases (400 °C,600 °C, 800 °C). When the temperature reaches 800 °C, the original fiber disappears and becomes particulate matter, causing many small holes. This may be a good way to increase the specific surface area. Measurements of carbon monoxide show that the gas-sensitivity test performs best at 600 °C calcination and a working temperature of 200 °C, with an amazing accuracy of 1 ppm. This result may be due to the unique geometry of the fibers.

### 2.2. Onefold Element-Doped $TiO_{2}$ Gas Sensors

Due to bandwidth gaps (3.0−3.2 eV), the ${\mathrm{TiO}}_{2}$ performance does not appear to be well considered. There are several ways to reduce bandgaps. During the doping process, the researchers found that different types of impurities exhibited different binding properties. For example, when inert metals are adulterated, the physical properties may change, which will help to improve the physical performance [64]. On the other hand, with different Fermi levels of nonmetallic or semiconductor particles doped, there are a broader range of applications [65]. The main reason is the reduction or change in the energy bandgap after doping, which belongs to a physical mechanism. The other is a chemical mechanism, which happens primarily through activation or catalysis to improve the sensitivity of the gas.

A $\mathrm{Au}-{\mathrm{TiO}}_{2}$ nanotube array for detecting ${\mathrm{SF}}_{6}$ decomposition gas was prepared by the deposition sedimentation method, and the tubular structure with a diameter of 25 nm was obtained. After Au doping in the nanotube array, the main shape of the pipeline is maintained, but the holes are covered with Au-atomic particles. Then, the performance of the gas sensor is discussed in detail. Firstly, the authors try to find out the most suitable working temperature in an operating range from 20 °C to 200 °C. The results show that the operating temperature is higher than 110 °C and the desorption rate is greater than the adsorption speed. Zhang [36] then tried to find out which gas reacted best; sensors working at optimum operating temperatures seemed to identify the response curves of ${\mathrm{SO}}_{2},{\;\mathrm{SOF}}_{2},{\;\mathrm{SO}}_{2}F_{2}{\;\mathrm{and}\;\mathrm{SO}}_{2}F_{2}$, ${\mathrm{SO}}_{2}F_{2}$ as compared to the best of the gas testing methods. Thus, the stability of the gas sensor was tested, and the results show that the Au-doped sensors have good stability. Finally, Zhang concluded that the Au-atomic addition of ${\mathrm{TiO}}_{2}$ can effectively reduce the operating temperature and increase gas sensitivity, which is one of the research objectives. Additionally, doping with precious metals mixed with silver through different methods was carried out by Haixin Liu et al. [25].They developed a quantum dot (QD) set of tiny mitochondrial quantum dots of titanium dioxide $\left({\mathrm{TiO}}_{2}\right)$ to impregnate QDs at low temperatures  (80 °C) with molecular beams of different concentrations of Ag(0−5%). The experimental results show that the QDs response of 3% doping is 6 times higher than that of unaerated ${\mathrm{TiO}}_{2}$ QDs in an ${\mathrm{NH}}_{3}$ atmosphere. At the same time, the gas sensor material can be coated on flexible substrates with excellent performance at room temperature. Additionally, for the detection of ammonia gas, Kaidi Wu et al. [26] were able to obtain the results by doping Ce, one of the rare-earth elements, into anatase ${\mathrm{TiO}}_{2}$ by using the microwave-assisted solvothermal method to generate nanocrystals, which achieved a response to the ${\mathrm{NH}}_{3}$ gas at room temperature when the doping concentration of Ce reached 0.43 at.%. The minimum detection concentration was 140 ppb, whereas the response/recovery speed, selectivity, and operational stability were improved. The authors also explain the change in resistance during gas detection. When the gas sensor is exposed to air at room temperature, free electrons are transferred from the conduction band of $\mathrm{Ce}-{\mathrm{TiO}}_{2}$ to the surface, producing adsorbed oxygen ions $O_{2\;\left(\mathrm{ads}\right)}^{-}$, which leads to an upward bending of the energy band and the formation of an electron-withdrawal layer at the interface, forming a Schottky barrier, at which point the gas sensor has a high resistance value. When the gas sensor is exposed to ${\mathrm{NH}}_{3}$ gas, the chemical reaction between the oxygen ions and the reducing ${\mathrm{NH}}_{3}$ molecules will release electrons, and this release of electrons makes the electron depletion layer thinner and the height of the Schottky barrier decreases, manifesting as a lower resistance value. Alexandra Teleki et al. [66] doped Cu elements in the same subgroup as Au and Ag into ${\mathrm{TiO}}_{2}$ by using flame combustion and found that the transition temperature from the anatase phase to the rutile phase was reduced to 400 °C when the doping amount was 5 at.%. After gas-sensitivity testing of the doped samples with CO (50−750 ppm), it was revealed that the response to different gas concentrations decreased when the working temperature was 400 °C. The recovery time was 10–15 s.

However, the results of Abdelilah Lahmar et al. [67] showed that the size of platinum particles was independent of the annealing atmosphere and the properties of Pt precursors. Additionally, Weerasak Chomkitichai et al. [68] allowed Pt loading on ${\mathrm{TiO}}_{2}$ by preparing hydrogen sensors through spin-coating Pt-loaded ${\mathrm{TiO}}_{2}$ films on ${\mathrm{Al}}_{2}O_{3}$ substrates using the flame-made method. Thanks to the spillover effect, the operating temperature was reduced to 300 °C when 2.00 mol% of Pt was loaded, at which time, the hydrogen concentration was 1 vol% and the response value was 470, and thus, the spillover effect was maximized. Although both Au and Pt are precious metals, the effects of doping can be quite different. This is not the only way in which Au has been affiliated, as Alireza Nikfarjam and Nahideh Salehifar [37] prepared fibers with a diameter of 80 nm by using the electrostatic spinning method and tested them with hydrogen. The best results were obtained with a response value of more than 10 to 75 ppm of hydrogen at 290 °C. Amazingly, when the hydrogen of the same concentration was tested under UV light at 360−380 nm, the response value exceeded 90 and the operating temperature decreased by about 100 °C. A 2.5-nanometer-thick gold layer was applied to the sensor surface by direct-current sputtering. The operating temperature was further reduced to 120 °C, and the sensor’s response to hydrogen under ultraviolet lamps of 400 nanometers reached 200. Similarly, the author explained the sensing mechanism of the ${\mathrm{TiO}}_{2}$ nanofiber sensor as a metal-oxide-film mechanism.

L. Aldon et al. [69] doped Sn into ${\mathrm{TiO}}_{2}$ and then applied it to lithium batteries. The mechanism of the lithium battery is based on the redox galvanic couple ${\mathrm{Ti}}^{4+}/\;{\mathrm{Ti}}^{3+}$, which works at 1.5 V. When Li is inserted into ${\mathrm{TiO}}_{2}$, the three-step mechanism of electrochemistry is followed. The first step is to insert the topology into ${\mathrm{Li}}_{x}{\mathrm{TiO}}_{2}\;(x<0.07)$, after which a small distortion occurs in the structure. Then, with the formation of ${\mathrm{Li}}_{0.5}{\mathrm{TiO}}_{2}$, the potential energy of 1.75 V can be described as a two-step mechanism. Last, but not least, the third step involves local insertions in lithium titanate ${\mathrm{Li}}_{0.5+x}{\mathrm{TiO}}_{2}$ alloys. In this step, the addition of tin will limit swelling, which means a good cycling ability of the compound. This is because the tin is located in the titanium crystalline structure of the anatase structure, and at the beginning of charge/discharge, it is reduced to ${\mathrm{Sn}}^{\mathrm{II}}$, which forms a passivation layer on the surface of particles. In the study of A. Teleki et al., they also doped with another element, Nb; ${\mathrm{TiO}}_{2}$ doped with 4 at.% Nb showed higher response values compared to ${\mathrm{TiO}}_{2}$ doped with Cu for CO (50−750 ppm) and also for ethanol at a minimum concentration of 25 ppm. In the experiments of Sukon Phanichphant [70], a similarly prepared method was used to dope the Nb elements, which were tested separately for ethanol and acetone gases. The 3 at.% Nb-doped sample had a response value of 31.7 and a response time of 1s for 400 ppm of ethanol at 400 °C and a response value of 13.0 and a response time of 33 s for 400 ppm of acetone gas at 400 °C. This response is because the size of ${\mathrm{Nb}}^{5+}\;\left(0.64\;Å\right)$ is similar to that of ${\mathrm{Ti}}^{4+}\;\left(0.605\;Å\right)$ and Nb can form a solid solution in the crystal structure of ${\mathrm{TiO}}_{2}$.

Single-layer graphene (SLG) has the purest carbon chemistry. The two-dimensional (2D) atomic structure maximizes the surface-to-volume ratio. The SLG’s tiny resistance value is very friendly to gas detection at a low power consumption and usually enables a significant reduction in operating temperature. If this can compensate for the higher operating temperature of ${\mathrm{TiO}}_{2}$, it will be a dramatic improvement in gas detection. As early as 2011, Qing Wang et al. [35] used the CVD method to grow ${\mathrm{TiO}}_{2}$ on SLG to form devices. Oxygen detection at room temperature and UV irradiation conditions were achieved. Unlike other works, S is not taken as the response value expression in this paper; instead $I_{D}=\frac{\Delta I}{I_{0}}$, where $I_{D}$ is the source-drain current, ΔI is the current change value, and $I_{0}$ is the initial current. The use of the detection of current changes has a linear electrical sensitivity, a condition essential in the detection of low concentrations (0.01%) of oxygen.

### 2.3. Other Substances Doped with $TiO_{2}$

Similarly, semiconductor materials are other common doping materials that are doped into ${\mathrm{TiO}}_{2}\;$ [71]. The findings suggest that harm to humans from harmful environments can be reduced regardless of the method used. Sometimes people need to dispose of hazardous waste, and sometimes they have to be careful when producing hazardous substances.

Chunrong Xiong and Kenneth J. Balkus, Jr. [71] used ${\mathrm{SnO}}_{2}$ to reduce the ${\mathrm{TiO}}_{2}\left(\mathrm{TDT}\right)$-type straight-chain nanofilament to improve the photodegradation of various dyes, rather than using pure ${\mathrm{TiO}}_{2}$ nanowires and P25 (25% rutile and 75% anatase). ${\mathrm{TiO}}_{2}$ photochemical activity is high, and photochemical corrosion stability is good. Chunrong Xiong and Kenneth J. Balkus, Jr. have carried out a lot of fruitful work to improve the efficiency of the photocatalytic net charge transfer. Then, they discovered that when ${\mathrm{SnO}}_{2}\;\mathrm{and}\;{\mathrm{TiO}}_{2}$, two semiconductor particles, are coupled, the photogenerated electrons will move to the conduction band of ${\mathrm{SnO}}_{2}$, and the holes that photons produced would accumulate in ${\mathrm{TiO}}_{2}$ valence bands. This is an effective method to restrain the recombination of the charge produced by light, and furthermore, the photo-induced charge rate is enhanced by Sn doping at the same time. Finally, it was demonstrated that the optimum atomic ratio was achieved when there was one Sn atom for every 10 Ti atoms. The best atomic ratio for nanowires of one Sn atom to every 10 Ti atoms can also be used for the deep mineralization of recalcitrant organic matter when its photodegradation performance is better than that of P25. Zinab H. Bakr et al. [72] used the same materials in solar cells. Zhaoyang Liu et al. [73] conducted the electrostatic spinning process by using side-by-side double spinners close to the ${\mathrm{TiO}}_{2}/\;{\mathrm{SnO}}_{2}$-component nanofibers to improve photocatalytic properties. In addition, many studies have demonstrated the excellent performance of ${\mathrm{TiO}}_{2}-\mathrm{Ag}-\mathrm{RGO}$ ternary materials in photocatalysis due to their high surface area to volume ratio [74].

**Figure 6:**
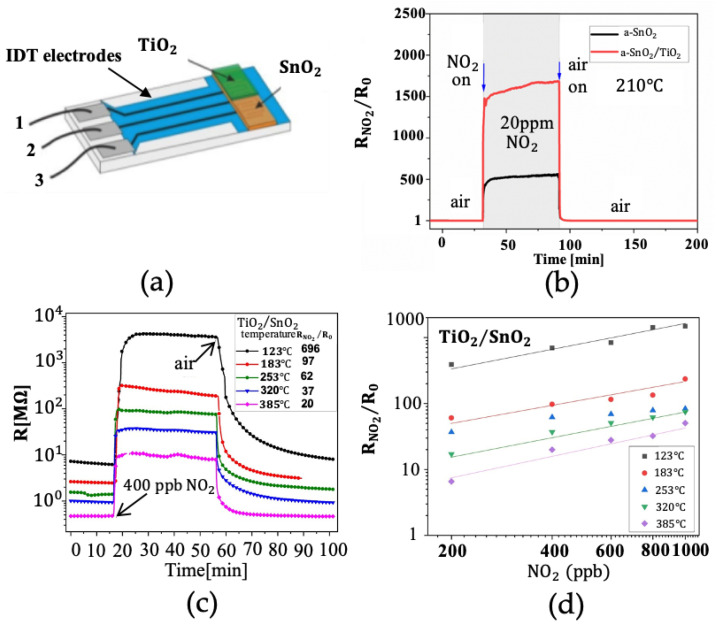
(**a**) Structure of the sensor; (**b**) the difference between a-${\mathrm{SnO}}_{2}$ and a-${\mathrm{SnO}}_{2}/{\;\mathrm{TiO}}_{2}$; (**c**) the electrical resistance changes at different temperatures in ${\mathrm{TiO}}_{2}/\;{\mathrm{SnO}}_{2}$ thin-film gas sensor; (**d**) ${\mathrm{TiO}}_{2}/\;{\mathrm{SnO}}_{2}$ thin-film gas-sensor response to a range of ${\mathrm{NO}}_{2}$ concentrations at different temperatures. Reprinted with permission from Ref. [41].

Piotr Nowak et al. [41] aimed to alert people early on to things that may be harmful to the body. Piotr Nowak and colleagues used magnetron sputtering (MS) to obtain a high sensitivity to ${\mathrm{NO}}_{2}$ in heterostructure thin films; the structure of the mechanism is illustrated in Figure 6a [41]. At the beginning of the work, the authors used MS to deposit a $200{\;\mathrm{nm}\;\mathrm{SnO}}_{2}$ substrate and then used the Langmuir–Blodgett technique to cover the thin and discontinuous ${\mathrm{TiO}}_{2}$ film. During the research, the authors found that amorphous ${\mathrm{SnO}}_{2}$ films have a better response to ${\mathrm{NO}}_{2}$ at a low concentration of 200 ppb, and the best $R_{{\mathrm{NO}}_{2}}/{\;R}_{0}$ reaction is obtained under the low working temperature of 120 °C. Thin-film heterostructure gas sensors have the advantage of selective detection of ${\mathrm{NO}}_{X}$ over $H_{2}$.

In the case of ${\mathrm{TiO}}_{2}/{\;\mathrm{SnO}}_{2}$, the resistance changes at the low concentration of ${\mathrm{NO}}_{2}$ to 400 ppb, and the gas sensor shows a different side to its normal perception; for example, the lower the operating temperature the better the performance. Figure 6b–d [41] shows the relationship between them. It is also mentioned that the gas $H_{2}$ reaction is smaller than the normal ${\mathrm{NO}}_{2}$ reaction, as the maximum reaction to $H_{2}$ reaches about 350 °C−400 °C, and the material can only be 18% improved by doping. The detection of hydrogen was also studied by Siti Amaniah Mohd Chachuli [75]. In their study, ${\mathrm{TiO}}_{2}-B_{2}O_{3}$ p-type heterojunction gas sensors were prepared, and response values of 1.44, 4.60, and 8.90 were measured at 300 °C for hydrogen concentrations of 100 ppm, 500 ppm, and 1000 ppm, respectively.

The same is true of Feng Li et al. [42], who doped ${\mathrm{TiO}}_{2}$ with ${\mathrm{SnO}}_{2}$ through electrostatic spinning. Interestingly, a parallel nozzle was used. After calcination, the surface is rough, and the nanofilament of ${\mathrm{TiO}}_{2}/\;{\mathrm{SnO}}_{2}$ is 206±17 nm in diameter. The nanofibers also show the excellent mechanical properties and selectivity of gas sensors, which are sensitive to ethanol and can be used for ethanol driving tests to avoid dangerous driving. In addition to ${\mathrm{SnO}}_{2}$ being used as the doped material, rare-earth metal oxides were also doped into ${\mathrm{TiO}}_{2}$ to modify their gas-sensitive properties. Porous ${\mathrm{TiO}}_{2}/{\;\mathrm{CeO}}_{2}$ nanoshells for CO gas were also fabricated using the flame combustion method by Bingcai Chen et al. [76]. From the energy-band engineering point of view, the authors found that the difference in the work function brought on by different materials would reduce the bandgap width and increase the carrier migration rate after the coupling of these two materials, thus improving the gas-sensitive performance. It is found experimentally that the oxygen-vacancy-rich nanoshells have an ultrafast response of 2 s and a fast recovery time of 6 s at 300 °C for a CO concentration of 500 ppm at ${\mathrm{TiO}}_{2}:{\mathrm{CeO}}_{2}=1:1$, which enables the material to detect CO gas at a minimum concentration of 500 ppb by enhancing the response to CO.

Jinniu Zhang et al. [27] doped titanium dioxide with different crystal phases and obtained a good response. In their work, new anatase@ rutile core@ shell nanoshells have been obtained via a two-step hydrothermal process, with a variety of thicknesses of the rutile crust surrounding the anatase core. When the thickness of the shell reaches 5 nm, the response of the heterojunction to various organic gases, especially ethanol, can approach 44. The author also tested the gas-sensitive response under different humidities and found that the gas-sensitive response test became less sensitive under the same condition with the increase in humidity. In terms of long-cycle stability, the gas-sensitive device showed stable results during 30 days of testing. Finally, the author analyzed and explained the mechanism of the sensor, and expressed his views on the mechanism from the aspects of work functions. Rather than focusing on the limitations of gas sensors, Patrick P. Conti et al. [77] discussed the type and rate of response to VOC gas, the electronic nose. For this reason, the author unusually added a conductive polymer as the doping material, and the resistance change in the nanofibers in the gas-sensitivity test was more obvious because of the synergistic effect of the combination and the formation of the junction. Finally, the author gives his own opinion on the mechanism of gas sensitivity, stating that the gas adsorption process should be physical and the interaction between them should be weak. The incorporation of polymers in the prepared sensors seems to be gaining acceptance by more and more researchers. G.J. Thangamani and S.K.Khadheer Pasha [43] synthesized nanofilms of ${\mathrm{TiO}}_{2}$ using the solution casting method, and the maximum sensitivity of the chemosensory of pristine ${\mathrm{TiO}}_{2}$ nanoparticles was 50.25% at 370 °C. Then, the authors added PVF to the solution for compounding with ${\mathrm{TiO}}_{2}$ fibers, which further reduced the operating temperature of the sensor to 150 °C for ${\mathrm{SO}}_{2}$ gas (600 ppm), with a sensitivity of 83.75% and an optimal ratio of $25\;\mathrm{wt}\%\;\mathrm{PVF}/{\;\mathrm{TiO}}_{2}$, an accelerated response time of 66 s, and increased stability after 60 days of testing.

In this section, the authors summarized the papers on ${\;\mathrm{TiO}}_{2}$-based gas-sensitive sensors that were undoped, single-element doped, multi-element doped, and those that incorporated DFT to explain the mechanism of experimental results. Selected experimental results related to response sensitivity are also summarized in Table 1 for a more intuitive comparison of these results.

## 3. DFT Calculation

Obtaining better results and breakthroughs often requires a lot of research time and the persistence of countless researchers to make gas sensors work better, but there is no doubt that it is often more difficult when we try to explore the mechanism of the reaction. In the last decade, the development of quantum chemical computational molecular simulations and density functional theory has allowed us to take a more microscopic view of the reaction process, which has attracted more and more people to participate in the research. First principles have helped us to take our research to a higher level from a physics perspective. First-principle computing has several attractive features. First, it can obtain the characteristics of a system without any experimental parameters [78], based on the general principles of quantum mechanics to complement experiments [79]. In the second place, it can be used as a prediction method for defect identification and characterization [80]. Third, it can be used as a prediction method for defect identification and characterization or to study the effects of geometric and electronic structures or interactions [81]. Finally, the density functional theory (DFT), DFT + ***U*** [82], DTF + ***U*** + ***J*** [23] and the hybrid function can allow us to obtain satisfactory results that are difficult to achieve in experiments. The characteristics of each step, when combined, are similar to conducting rigorous experiments, which is why more and more researchers are trying to use the first-principle calculation.

In the microscopic field of theory, physicists use an equation called the wave functions to describe the states of particles, which is generally expressed as Equation (7):

(7) Ψ=Ψ|H|Ψ

where Ψ is the wave function, and H is the Hamiltonian. With the continuous efforts of scientists, the emergence of density functional theory has solved this problem in time. The main idea is to replace the wave function with the charge density, which was proposed by Thomas and Fermi as early as 1927 [83]. The Hohenberg–Kohn (H-K) [84] theorem was later developed, and the Hamiltonian quantity was written based on the Thomas–Fermi theory as H=T+U+V [84], which contains the kinetic energy and potential energy describing the particle. However, the problem of electron density is still not well handled. Then, the Kohn–Sham (K-S) [85] equation appeared to solve this problem, giving the total energy. At this time, the density functional theory has a more complete theoretical basis, and only part of the exchange-correlation energy functional and potential remains unsolved. As many as hundreds of interaction association approximations, such as LDA [86], GGA [87], and PBE [88], are approximate solutions for the interaction association energy and potential energy; therefore, these approximate solutions determine the accuracy of the calculation.

### 3.1. The DFT Calculation Combined with Experiment

First-principle calculation can be used to perform many simulations, including some dangerous biological experiments, without any side effects. However, as mentioned earlier, doping is an effective way to improve the performance of semiconductor gas sensors. A doped semiconductor gas sensor has been widely used in first-principle calculation [89]. For example, Mingjia Zhang et al. [90] found a very good agreement by comparing the bonding in the XPS test results with the bonding in the calculated results. At the same time, the first principle also shows its advantages, namely, it saves more resources but makes It possible to obtain richer information about the system. Hua Gui Yang et al. [91] used the first principle to predict and prepare 47% (0 0 1) of the fluoroterminated surfaces. In addition, fluorine-containing surfaces can be easily removed by heat treatment to recover nonfluorine surfaces without altering the crystal structure. Xing Gao et al. [4] wrote about similar results.

Compared with STM images, it is found that Nb doping has a stronger attraction to conductive bands and self-trapped electrons than the Ta dopant, and HSE06 is more suitable for Nb- and Ta-doped ${\mathrm{TiO}}_{2}$. To clarify the oxygen adsorption model utilizing calculations, Jiang-Wei An and Gui-Chang Wang [92] derived from the Cu-doped ${\mathrm{TiO}}_{2}$ study using DFT calculations that the difference in the concentration of unsaturated dislocated oxygen $\left(O_{2c}\right)$ at different concentrations reflects the results of the ${\mathrm{TiO}}_{2}$ surface energy, and that a higher surface energy corresponds to the higher concentration of unsaturated dislocated oxygen $\left(O_{2c}\right)$ and a better catalytic performance. Metal oxide can improve the gas-sensing performance [38]. The surface of ${\mathrm{TiO}}_{2}$ is modified with a psychoactive oxide cluster by Michael Nolan et al. [93]. In Lili Wang’s [39] work, the cladding structure of the $\mathrm{NiO}/{\;\mathrm{TiO}}_{2}$ HNFs was prepared. The first-principle model is then used to explore three different types of gas $\left(\mathrm{CO},{\;H}_{2}S,{\;\mathrm{and}\;H}_{2}\right)$. The experimental and calculated data show that the gas sensor has good selectivity to carbon monoxide with a high sensitivity and low detection limit (1 ppm). The work of Juan Liu [94] and others is similar. The adsorption model was calculated theoretically and the mechanism was analyzed in detail. The results show that N-doping ${\mathrm{TiO}}_{2}$ particles have strong activity in surface adsorption to remove small molecules of toxic and harmful human gases. The results of Patricia Lopez-Caballero [95] showed that different results can validate calculations of precise control patterns using noble metal doping. In addition, through computer technology, the principle and processes of gas adsorption reactions have become clearer. CO was also used as a detection gas in the results of the study by Utkarsh Kumar et al. [44]. The researchers prepared ultrafine ${\mathrm{TiO}}_{2}/\;\mathrm{PbSnS}$ films using the continuous ion-layer adsorption and reaction (SILAR) method, and the addition of PbSnS changed the forbidden band of ${\mathrm{TiO}}_{2}$ from 3.3 eV to 2.3 eV. The response value of 8.3 was detected at room temperature for a concentration of 60 ppm of CO, the lowest detection limit for carbon monoxide was down to 3.89 ppm, which is given by an alternative expression for the response values shown in Equation (8) [44,96]. It is worth noting that Equations (5) and (8) can equally describe the sensitivity response values. For n-type semiconductors, it is the resistance of the device in the gas being detected that is used as the divisor phase of the equation, with the gas in the air or the magnitude of the change in device resistance as the divisor. For p-type semiconductors, the resistance in the air is used as the divisor phase of the equation, and the resistance in the gas being detected or the change in resistance is used as the divisor phase. Both Equations (5) and (8) can be used as the formula for response sensitivity.

(8) $$\mathrm{SR}\left(p-\mathrm{type}\right)=\frac{R_{\mathrm{gas}}-R_{\mathrm{air}}}{R_{\mathrm{air}}},\mathrm{SR}\left(n-\mathrm{type}\right)=\frac{R_{\mathrm{air}}-R_{\mathrm{gas}}}{R_{\mathrm{gas}}}$$

It was found by theoretical calculations that the CO drifts toward the Sn atom during the process of being adsorbed. In addition, since the detection results of gas sensors are usually related to the humidity in the environment, the researchers also included water molecules in the calculations to analyze the real situation during actual detection. The adsorption energy was calculated to be −13.6 KJ/mol for carbon monoxide without water molecules, and then, the adsorption energy became 25.7 KJ/mol when water molecules were added to the system; thus, the authors obtained that the presence of water molecules did not affect the detection of CO by the sensor.

**Figure 7:**
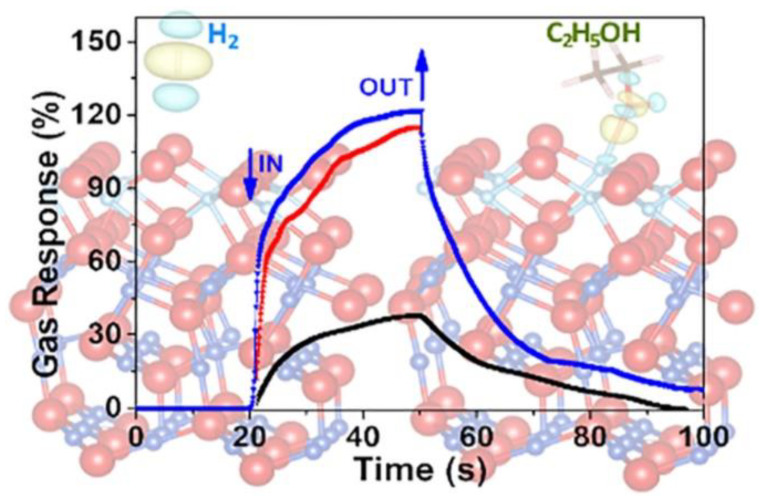
Simulations are a good way to interpret actual test results. Where are the dynamic response results of 20 nm thickness of CuO/Cu_2_O at 250 °C (black line), 300 °C (red line), and 350 °C (blue line) to 100 ppm ethanol. Reprinted with permission from Ref. [97]. Copyright 2021, American Chemical Society.

Oleg Lupan et al. [97] prepared mixed-phase heterostructures of titanium dioxide/cooperate/copper oxide $\left({\mathrm{TiO}}_{2}/\mathrm{CuO}/{\;\mathrm{Cu}}_{2}O\right)$ by using the spray sputter annealing method. The formation of binary heterojunctions $\mathrm{CuO}\;\left(111\right)/{\;\mathrm{Cu}}_{2}O\;\left(111\right)$ and ternary heterojunctions ${\mathrm{TiO}}_{2}\;\left(111\right)/\mathrm{CuO}\;\left(111\right)/{\mathrm{Cu}}_{2}O\;\left(111\right)$ and their reactions to three gases, $H_{2}$, $C_{2}H_{5}\mathrm{OH}$, and $n-C_{4}H_{9}\mathrm{OH}$, were also simulated by first-principle calculations, and some of the results of the simulations are shown in Figure 7 [97]. The calculated results show that the value of the work function increases with the number of heterojunction components, which is consistent with the conclusion that the sensitivity of gas-sensing heterojunctions varies. $H_{2}$ released the minimum adsorption energy on the surface of the nanodevice and $C_{2}H_{5}\mathrm{OH}$ released the maximum adsorption energy on the surface, which is consistent with the gas selectivity obtained from gas-sensing tests, both reflecting a high selectivity for ethanol. This indicates that an adsorption model compatible with the surface structure is an essential and effective means to explain the binding energy variation.

Bharat Sharma et al. [51] looked at machine gas detection as well and found that ultrahigh sensitivity was important because diabetics exhaled more than 1.8 ppm of acetone, a level that exceeded 0.3 to 0.9 ppm in normal subjects. The authors prepared n−n, ${\mathrm{TiO}}_{2}-{\mathrm{SnO}}_{2}$ heterostructures using a combination of pulsed laser deposition (PLD) and reactive magnetron sputtering (RMS) methods and confirmed the successful preparation of the heterojunctions by verifying the XRD test results using the Scherrer equation [40]:

(9) $$D=\frac{0.89\lambda }{\beta \cos\theta }$$

where D= crystallite size, λ= wavelength of XRD (Å), β= full width at half-maximum of the diffraction peaks, and θ= Bragg angle. The optimum operating temperature of 300 °C and the minimum detection limit of 0.02 ppm were obtained via a gas-sensitivity test, and the results were 12 times better than those of the ${\mathrm{TiO}}_{2}$ sensor. Subsequently, the author also conducted a stability test, which showed stabilization at 300±0.5 ppm for 60 days. In the DFT calculation section, the formation energy at the heterojunction interface was calculated by Equation (10) [51]:

(10) $$\Delta E\left(\frac{{\mathrm{TiO}}_{2}}{{\mathrm{SnO}}_{2}}\right)=\frac{E\left({\mathrm{SnO}}_{2}\right)+E\left({\mathrm{TiO}}_{2}\right)-E\left(\frac{{\mathrm{TiO}}_{2}}{{\mathrm{SnO}}_{2}}\right)}{S}$$

where S is the interface area. Then, the adsorption energy of sensing can be calculated as [51]:

(11) $$E_{\mathrm{ads}}=E_{\mathrm{surf}+\mathrm{mol}}-E_{\mathrm{surf}}+E_{\mathrm{mol}}$$

where $E_{\mathrm{surf}+\mathrm{mol}}$ is the total surface energy of maximum adsorption, $E_{\mathrm{surf}}$ is the optimum energies of surface models (110) before the adsorption of gas molecules, and $E_{\mathrm{mol}}$ is the isolated gas molecule. The results were −1.02 eV and −1.89 eV. The authors then found a link between the results and DFT calculation simulations, which was expressed as [51]:

(12) $$\Delta R\propto e^{-\frac{e\Delta V_{b}}{\mathrm{kT}}}$$

where ΔR is the value of the change in sensor resistance, $\Delta V_{b}$ is the reduction of the potential barrier, κ is the Boltzmann constant, and T is the absolute temperature. The consistency and coherence of experiments and calculations are also demonstrated. This shows that simulation is an effective method for simulation experiments and gives a clear reaction mechanism.

### 3.2. More In-Depth Theoretical Calculations

Joseph Muscat et al. [98] studied the ${\mathrm{TiO}}_{2}$ elastic properties, compressibility, and phase transitions of various polymorphisms with the first principles. Sergej Krylow and Martin E. Garcia [99] studied the phase transition of ${\mathrm{TiO}}_{2}$ at different temperatures and that was laser-induced by the atomic starting point. Some research on the infrastructure of crystals is important for guidance and reference. Most of the anatase ${\mathrm{TiO}}_{2}$ crystals are composed of more than 94% stable (1 0 1) facets and the other portion are more reactive and differential, whereas the rest are more active (0 0 1) [100]. This also means that it is necessary to know what is happening in which environment, whether in experiments or computer simulations, as understanding the mechanism of the reaction is helpful. In addition, different aspects may expose different quantities of dangling bonds, which may lead to different catalytic activities. Wen Zeng [100] calculated the model of oxygen adsorption on the surface of anatase ${\mathrm{TiO}}_{2}\;\left(1\;0\;1\right)\;\mathrm{and}\;\left(0\;0\;1\right)$ using the first principle, and the result is that oxygen molecules in the ${\mathrm{Ti}}_{5C}$ position on the (0 0 1) surface can make more oxygen atoms adsorbed on the surface than the ${\mathrm{Ti}}_{5C}$ position on the (1 0 1) surface, thus forming more adsorbed oxygen sites and improving the sensor performance, which means that when the prepared material has more anatase ${\mathrm{TiO}}_{2}\;\left(0\;0\;1\right)$ surfaces, the more likely it is to improve the performance of the gas sensor. First-principle calculations, along with the results of the study, also convey another message: that in some aspects, first-principle calculations are fully capable of being more detailed and comprehensive.

To verify the accuracy of the results, James A. Quirk et al. [101] characterized the boundary and double boundary of Σ3 (112)  and  Σ1 (110); twin boundaries (TBs) are described using transmission electron microscopy (TEM) and compared with the electronic structure calculated by the first principle, and a good structural consistency is obtained. This is common, but is strong evidence of accuracy.

**Figure 8:**
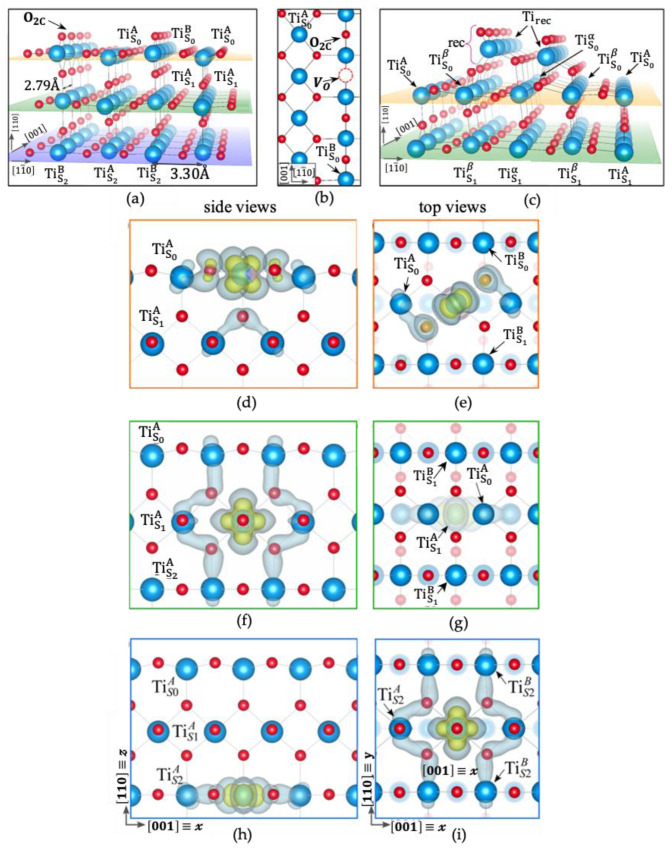
Facet structures of (110). Front view (**a**) of the pristine (1×1) phase; top view (**b**) of the reduced (1×1) phase; and front view (**c**) of the (1×2) reconstruction. Polaron charge density. The side and top views of the ${\mathrm{Ti}}_{S0}^{A}$ polaron (**d**,**e**), ${\mathrm{Ti}}_{S1}^{A}$ polaron (**f**,**g**), and ${\mathrm{Ti}}_{S2}^{A}$ polaron (**h**,**i**) are shown. The inner and outer isosurfaces represent different levels of the charge density of the polaronic states. Fade spheres represent deeper atoms in top-view images; S0 and S1 atoms not shown in panel (**i**). Reprinted with permission from Ref. [102]. Copyright 2022, American Physical Society.

Michele Reticcioli et al. [102] also carried out an in-depth study of its surface. Michele Reticcioli found that charge capture, polaron formation, and kinetic processes can be simulated using the first-principle technique, as shown in Figure 8a–c [102]. A large number of polarons are accommodated by the structural reconfiguration of the system, and then, the interaction between polarization poles and the polarized airspace is investigated, as shown in Figure 8d–i [102]. Finally, it is concluded that the behavior of the ${\mathrm{TiO}}_{2}$ small polaron can be explained, and it is also determined that a site-specific polaron may affect the interaction of adsorbents and may play a key role in catalysis. By doing so, the principles of action are so clearly expressed that research can be purposefully pushed forward.

Bandwidth changes the most quickly after stimulant use because the first principle can show changes before and after doping [103]. The bandgap maps obtained by doping with different elements are different from those given by Yi Wu [104]. Through the Nd-doped, C-doped, and Nd−C-codoped calculations, it is found that visible light can excite more electrons at the energy level of an empty Nd 4f than Ti 3d can in orbit. Xiaoxing Zhang et al. [105] used the first principle to analyze the adsorption mechanism of ${\mathrm{SF}}_{6}$ and its decomposed components (${\mathrm{SO}}_{2},{\mathrm{SOF}}_{2},{\mathrm{SO}}_{2}F_{2}$) in the N−F-codoped ${\mathrm{TiO}}_{2}$ system. The results show that the gas-sensing response of ${\mathrm{SO}}_{2}$ is better than the other two decomposition components, indicating that N−F-codoped ${\mathrm{TiO}}_{2}$ has good selectivity to ${\mathrm{SO}}_{2}$. Takenori Yamamoto and Takahisa Ohno [106] not only calculated the energy band, but also showed partial charge densities for the occupied state.

**Figure 9:**
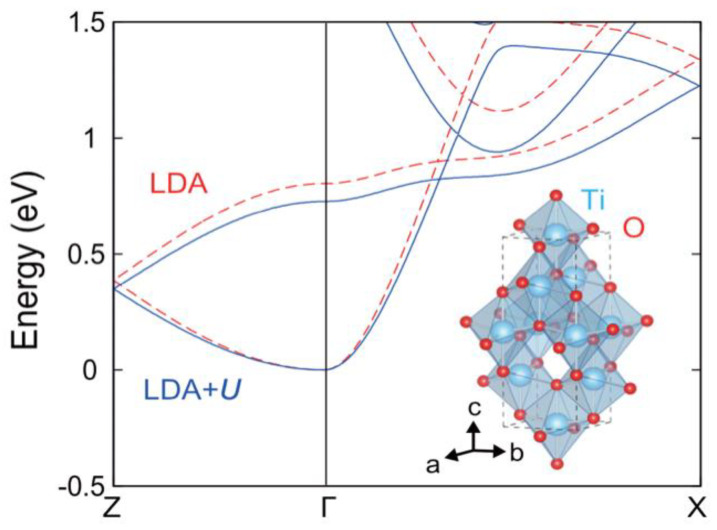
The contrast of ${\mathrm{TiO}}_{2}$ calculation results via LDA and LDA + ***U***. Reprinted with permission from Ref. [107]. Copyright 2022, American Physical Society.

In the early stage of the crystal system, the direct use of function calculation results may deviate from the experimental values. Therefore, a solution of DFT + ***U*** or other hybrid functions is proposed. Youngho Kang et al. [107] used DFT + ***U*** to calculate the electron–phonon interaction and transmission in anatase ${\mathrm{TiO}}_{2}$. Figure 9 [107] compares the different results. The image is drawn along the high symmetry lines of the Brillouin zone (BZ), where the minimum conductivity band is set as a reference frame for the LDA (red dashed line) and the LDA + ***U*** (blue solid line). The lower right corner gives the quadrilateral crystal structure with blue atoms of titanium and red atoms of oxygen. To better describe the structural properties, the on-site Coulomb energy U=3.3 eV is used, and the results are renormalized. Finally, the authors write that the formation of large polarons plays an important role in ${\mathrm{TiO}}_{2}$ electron-mobility analysis, and velocity regularization is the key to modifying the scattering time to 20%  of the moving rod while considering the electron–phonon coupling through the multibody perturbation theory.

Okan K. Orhan and David D. O’Regan [108], based on the first principle by combining the DFT + **U** and Hund’s **J** corrections with parameters calculated using linear response theory based on an extended first principle, found that this method was able to predict the bandgap more accurately, giving a maximum error of less than 0.03 eV in the calculated bandgap values compared to the experimental values, and then, they tried the +U treatment for only the Ti 3d orbitals and the + U treatment of the 2p orbitals of O, also obtaining closer bandgap values. The results also show that appropriate calculation parameters are necessary to have reliable results. The density of states (DOS) can be seen as a visualization with a band structure, whereas the partial density of states (PDOS) can show the details of the point-to-point connection. Le Huang et al. [109] researched interfacial passivation of four different cushioning materials. Naoki Nagatsuka et al. [110] added the +U=4 eV adjustment to their calculation system to adjust the bandgap to 3.2 eV. The difference is that N. Nagatsuka et al. may be more focused on the reactions that occur at the surface, leading to a more in-depth fundamental explanation of these reactions, and the authors clarified that hydrogen at the surface of anatase ${\mathrm{TiO}}_{2}\;\left(1\;0\;1\right)$ generates excess electrons near the oxygen vacancy, thus forming an off-domain electronic state, which is in contrast with the excess electrons at the oxygen vacancy of the rutile ${\mathrm{TiO}}_{2}$ surface that forms a midgap state at the Fermi energy level. This contrasts with the formation of a midgap state at 0.8 eV below the Fermi energy level in rutile ${\mathrm{TiO}}_{2}$. The authors also mentioned that in their previous study, the presence of H was detected on the surface of anatase ${\mathrm{TiO}}_{2}$ at 300 K by nuclear reaction analysis, and the H content decreased when the temperature was increased to 500 K. When heated to 700 K, no H was detected at all.

In addition to the relationship between polarons and electrons, the first-principle calculations can also study the surface or defects of the ${\mathrm{TiO}}_{2}$ surface.

**Figure 10:**
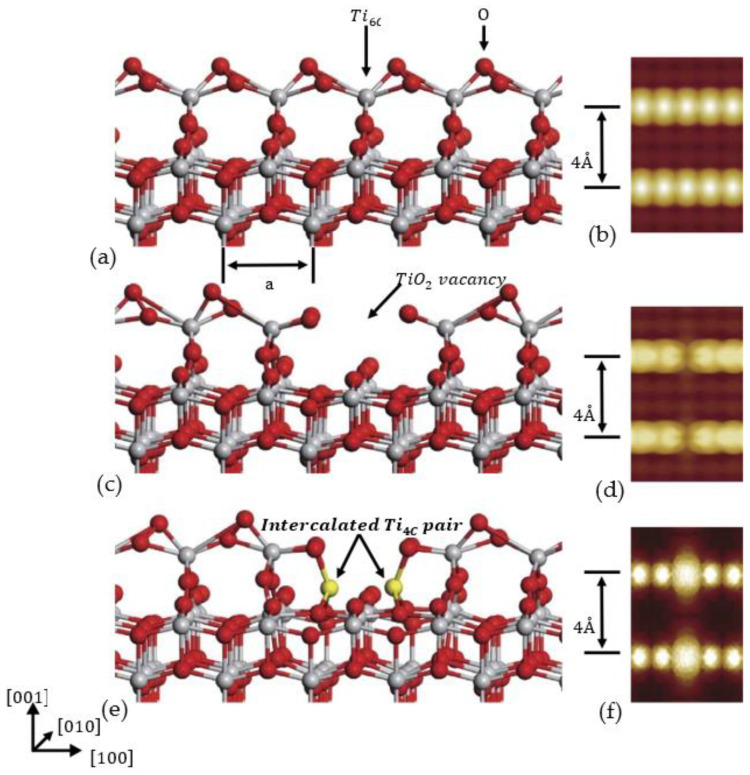
The models of calculation: (**a**,**b**) show the perfect ${\mathrm{TiO}}_{2}$ structure; (**c**,**d**) show the dark defect (${\mathrm{TiO}}_{2}$ vacancy); (**e**,**f**) show the bright defect (intercalated Ti pair). The sixfold-coordinated Ti (${\mathrm{Ti}}_{6C}$) at the ridge and fourfold-coordinated $\mathrm{Ti}\;({\mathrm{Ti}}_{4C}$) at the defect site are labeled in (**a**) and (**e**)**.** Reprinted with permission from Ref. [111].

The surface of anatase ${\mathrm{TiO}}_{2}\left(0\;0\;1\right)-\left(1\times 4\right)$ was studied by microscopy and spectral analysis, and two types of intrinsic point defects were identified by Yang Wang [111]. The results showed that only the surface of the ${\mathrm{Ti}}^{3+}$ defect showed greater activity, and if it was perfect, it would oxidize completely, leading to low activity. Therefore, when the temperature is room temperature, ${\mathrm{TiO}}_{2}\;\left(0\;0\;1\right)-\left(1\times 4\right)$ surfaces cannot even adsorb on water. However, at 80 K, the Ti-rich point defect will have active sites of $H_{2}O$ and $O_{2}$. Using the STM image shown in Figure 10 [111] as an example, the mathematical model was established and studied by Wang et al. It is found that under the control of STM’s advanced technology, the two kinds of defects can be transformed into each other, indicating that they have the same basic structure. More importantly, at low temperatures, Ti-rich defects are probably the most active site for $H_{2}O$ and $O_{2}$ at the ridge on the reduced surface. It is well suited for experimentation. Philip J. D. Lindan and N. M. Harrison [112] explain a similar problem from another angle.

It is undeniable that two-dimensional (2D) materials are gaining more and more importance in some fields as researchers continue to refine and develop their research on materials [78]. Additionally, DFT calculations, with their convenience and safety, allow researchers to perform experiments that simulate some very demanding experimental environmental conditions. ${\mathrm{SF}}_{6}$ is widely used in transmission and distribution equipment for insulation and arc extinguishing. Although ${\mathrm{SF}}_{6}$ is more stable, it inevitably reacts with water molecules and oxygen in the air in microreactions to form ${\mathrm{SO}}_{2}F$, ${\mathrm{SOF}}_{2}$, and ${\mathrm{SO}}_{2}F_{2}$. In the article, Hao Sun [20] performed calculations by building a monolayer GeSe model as the overall model base with ${\mathrm{TiO}}_{2}$ as the dopant. Firstly, the adsorption energy of the monolayer GeSe for ${\mathrm{TiO}}_{2}$ was calculated as −3.356 eV, and then, the adsorption energy for other gases was calculated separately with the presence or absence of ${\mathrm{TiO}}_{2}$ as the comparison difference. The adsorption energies of $H_{2}S$, ${\mathrm{SO}}_{2}$, ${\mathrm{SOF}}_{2}$, and ${\mathrm{SO}}_{2}F_{2}$ were −0.412 eV, −0.667 eV, −0.431 eV and −0.3334 eV, respectively, in the absence of ${\mathrm{TiO}}_{2}$, and changed to −0.876 eV,−1.023 eV,−0.6 eV,  and −0.36 eV in the presence of ${\mathrm{TiO}}_{2}$ adsorption energy. With this result, it is obvious that the presence of ${\mathrm{TiO}}_{2}$ helps the system to obtain a larger adsorption energy, which indicates that the doping of ${\mathrm{TiO}}_{2}$ is successful in improving the gas-sensitive response of the monolayer GeSe to the ${\mathrm{SF}}_{6}$ decomposition gas. Additionally, the authors gave the conductivity (σ) of the gas adsorption system for better integration with the experiment, which can be expressed by Equation (13) [113]:

(13) $$\sigma \propto e^{-\frac{E_{g}}{2\mathrm{kT}}}$$

where $E_{g}$ is the energy gap, expressed by the following equation:

(14) $$E_{g}=\left|E_{\mathrm{LUMO}}-E_{\mathrm{HOMO}}\right|$$

which is by Qingfang Zhang et al. [113], who interpreted $E_{g}$ as an energy gap between the molecular orbitals; the highest occupied molecular orbital (HOMO) and the lowest occupied molecular orbital (LUMO) were calculated to assess the electrical conductivity of the adsorption system by using Equation (13). κ is the Boltzmann constant, and T is the working temperature. Using this equation, the authors found that the results obtained from the calculations were in good agreement with the results given by the experiment. The authors also calculated the theoretical recovery time of the gas, given by Equation (15):

(15) $$\tau =v_{0}^{-1}e^{\frac{-E_{\mathrm{ads}}}{\kappa_{B}T}}$$

which also obtained the same trend as the experimental results. Finally, Sun et al. determined that the addition of ${\mathrm{TiO}}_{2}$ makes GeSe have a better adsorption performance on the decomposition gas of ${\mathrm{SF}}_{6}$.

Graphene, with its unique two-dimensional structure and large specific surface area, has gradually become a hot spot for sensor research [114]. Zhang et al. used DFT calculations to simulate the adsorption of ${\mathrm{SF}}_{6}$ decomposition gas by, for example, three different metal oxides, ${\mathrm{TiO}}_{2}$, ${\mathrm{Fe}}_{2}O_{3}$, and NiO. The final result was that the bandgap value of ${\mathrm{TiO}}_{2}$-doped graphene decreases to 0.38 eV and has the best adsorption effect on ${\mathrm{SO}}_{2}F_{2}$. Although the adsorption of ${\mathrm{SO}}_{2}F_{2}$ by ${\mathrm{TiO}}_{2}$-doped graphene is physical adsorption, the hybridization of electronic orbitals and the filling of electronic states lead to the migration of the conduction and valence bands. The authors speculate that among the three different metal-oxide dopings, ${\mathrm{TiO}}_{2}$ doping is most likely to achieve the desired sensing effect.

In this section, the authors provided a brief overview of the theoretical basis of DFT calculations for ${\mathrm{TiO}}_{2}$, for instance, integrating the theoretical calculations with experiments, which was followed by a concise review of the more in-depth theoretical calculations.

## 4. Conclusions

In general, the excellent advantages of ${\mathrm{TiO}}_{2}$ in terms of nontoxicity, low cost, and high stability in harsh environments have attracted researchers to investigate it more intensively, not only in terms of experimental studies, but also in theoretical studies. The academic potential of ${\mathrm{TiO}}_{2}$ and its application potential in gas sensing and photocatalysis, alongside the development of experimental techniques and conditions, now allow us to try a multitude of new and efficient cascade methods, which can achieve a better sensor performance. However, if we simply improve our experimental results by doing more experiments, it will be prohibitively expensive. Undeniably, some basic research has been conducted over a long period of time with many excellent results, laying a solid foundation for future research, but also revealing the problems that still exist and areas for improvement, which is why more and more studies include computational methods such as DFT. The increasing proportion of theoretical calculations in research requires researchers to have a new understanding of integrated experimental and theoretical approaches. Therefore, more microscopic-level information is necessary for the research process. This will help us to get closer to the experimental mechanism of the ${\mathrm{TiO}}_{2}$ gas sensor in each step of the process and closer to the truth, rather than simply referring to the experimental results. Higher accuracy and better experimental conditions promote the research and development of sensor technology. The increase in theoretical calculations allows researchers to analyze experiments faster and more safely, improving them from a microatomic perspective and in a targeted manner, and the researchers can obtain clarified results. This review started with an introduction to the properties and applications of ${\mathrm{TiO}}_{2}$, and then, the authors presented the experiments familiar to many researchers in the second part of this review, which was followed by the review of different methods and types of doped ${\mathrm{TiO}}_{2}$ gas sensors, reaction mechanisms, and how to combine some of the theoretical calculations with experimental results. In the future, we will undoubtedly use research methods of experimental and theoretical calculations in parallel, which will not only make up for the shortcomings around the clarification of experimental chemical reaction mechanisms, and the more microscopic and essential physical reaction mechanisms that are not known, but also will help test the results of some theoretical studies. In the future, theoretical calculations will be integrated into experiments, which will allow experiments and theoretical calculations to be coupled in greater depth, thus providing a microlevel analysis of the nature of experimental phenomena.

## Figures and Tables

**Table 1:** The performance of some ${\mathrm{TiO}}_{2}$-based gas sensors in this paper.

Materials/ Structure	Synthesis Methods	Detecting Gases/ Concentrations	Response (S)	Temperature (°C)	Response/ Recovery Time	Ref./ Year
${\mathrm{TiO}}_{2}$/nanoparticle	Flame spray pyrolysis	Isoprene/acetone (1–7.5 ppm)/ethanol/CO	NA	500 °C	Acetone: 2–3 s/144 s (1 ppm), 302 s (7.5 ppm)	[19]/ 2006
${\mathrm{TiO}}_{2}$/thin film	DC magnetron sputtering	${\mathrm{NH}}_{3}$/500 ppm	NA	250 °C	90 s/110 s	[33]/ 2007
polyaniline–titanium dioxide (PANI/${\mathrm{TiO}}_{2}$)/thin film	In situ chemical oxidation polymerization	${\mathrm{NH}}_{3}$/47 ppm	$S=\frac{R_{g}-R_{a}}{R_{a}}$	25 °C	5 s/69 s	[34]/ 2007
			2.33			
$\mathrm{LiCl}-{\mathrm{TiO}}_{2}$/nanofiber	Electrostatic spinning	NA	NA	25 °C	3 s/7 s	[31]/ 2008
			1000			
${\mathrm{TiO}}_{2}$-graphene/thin film	Chemical vapor deposition	$O_{2}/\begin{array}{c} \;\mathrm{Full}\;\mathrm{concentration}\\ \;\mathrm{range}\;\left(5\%–100\%\right) \end{array}$	$I_{D}=\frac{\Delta I}{I_{0}}$	Room temperature	130 s/260 s	[35]/ 2011
			3.65 (5% $O_{2}$)			
$\mathrm{Au}-{\mathrm{TiO}}_{2}$/nanotubes	Deposition sedimentation	${\mathrm{SF}}_{6}$ decomposition gas (${\mathrm{SO}}_{2}F_{2}/{\mathrm{SOF}}_{2}/{\mathrm{SO}}_{2}$)/50 ppm	$S=\frac{R-R_{0}}{R_{0}\;\times \;100}$	110 ℃	NA	[36]/ 2014
			$74.6{\%\;\mathrm{to}\;\mathrm{SO}}_{2}\frac{{\mathrm{TiO}}_{2}}{19.95}{\%\;\mathrm{to}\;\mathrm{SO}}_{2}F_{2}\;\left(\mathrm{Au}-{\mathrm{TiO}}_{2}\right)$			
${\mathrm{TiO}}_{2}$/Au-${\mathrm{TiO}}_{2}$/nanofiber/nanofiber@ nanofilm	Electrostatic spinning	$H_{2}:25\;\mathrm{ppm}\;\left(\mathrm{without}\;\mathrm{UV}\right)/5\;\mathrm{ppm}\;\left(\mathrm{with}\;\mathrm{UV}\right)$	$S=\frac{R_{\mathrm{air}}}{R_{\mathrm{gas}}}$	$H_{2}$:290 °C (without UV)/190 °C (with UV)	$H_{2}:12.3\;s/22.5\;s\;$(without UV) | 2 s/6.9 s (with UV)	[37]/ 2015
			10.1 (without UV)/96 (with UV)			
${\mathrm{TiO}}_{2}/\mathrm{SiC}$/core–shell hierarchical	Hydrothermal method	Acetone/100 ppm	$S=\frac{R_{\mathrm{air}}}{R_{\mathrm{gas}}}$	450 °C	1 s/NA	[38]/ 2018
			19.2			
$\mathrm{NiO}/{\mathrm{TiO}}_{2}/\mathrm{HNF}$ ultrafine NiO nanoparticles in ${\mathrm{TiO}}_{2}$	Electrostatic spinning	CO/1 ppm	$S=\frac{\Delta R}{R_{0}}$	Room temperature	10.0 s/12.5 s	[39]/ 2018
			1.02			
$\mathrm{Au}-{\mathrm{TiO}}_{2}$/QDs	Convenient hydrolysis method	${\mathrm{NH}}_{3}/10\;\mathrm{ppm}$	$S=\frac{R_{\mathrm{air}}}{R_{\mathrm{gas}}}$	Room temperature	150 s/600 s	[25]/ 2019
			25.1			
$\mathrm{Co}-{\mathrm{TiO}}_{2}$/nanotube	One-step anodization and immersion method	$H_{2}S/50\;\mathrm{ppm}$	$S=\frac{R_{\mathrm{air}}}{R_{\mathrm{gas}}}$	300 °C	14 s/4 s	[40]/ 2019
			199.16			
Anatase@ rutile ${\mathrm{TiO}}_{2}$/core@ shell	Two-step hydrothermal method	$C_{2}H_{5}\mathrm{OH}/100\;\mathrm{ppm}$	$S=\frac{R_{\mathrm{air}}}{R_{\mathrm{gas}}}$	270 °C	NA	[27]/ 2020
			*8.2*			
${\mathrm{SnO}}_{2}$/${\mathrm{TiO}}_{2}$ nanoheterostructures	Magnetron sputtering and Langmuir–Blodgett technique	${\mathrm{NO}}_{2}/400\;\mathrm{ppb}$	$S=\frac{R_{\mathrm{air}}}{R_{\mathrm{gas}}}\;$	123 °C	62 s/42 s	[41]/ 2020
			696			
${\mathrm{TiO}}_{2}//{\;\mathrm{SnO}}_{2}$ Janus nanofiber	Electrostatic spinning	$C_{2}H_{5}\mathrm{OH}/10\;\mathrm{ppm}$	$S=\frac{R_{\mathrm{air}}}{R_{\mathrm{gas}}}$	368 °C	8 s/13 s	[42]/ 2020
			7< S < 8			
PVF/${\mathrm{TiO}}_{2}$/nanocomposite films	Solution casting	${\mathrm{SO}}_{2}/600\;\mathrm{ppm}$	$S=\frac{R_{\mathrm{gas}}-R_{\mathrm{air}}}{R_{\mathrm{gas}}}\;\times \;100$	370 °C (not compounded)/150 °C (after compounding)	66 s/107 s	[43]/ 2021
			50.25% (not compounded)/83.75% (after compounding)			
${\mathrm{TiO}}_{2}/\mathrm{PbSnS}$/film	Successive Ionic Layer Adsorption and Reaction (SILAR)	$\mathrm{CO}\;60\;\mathrm{ppm}/{\mathrm{NO}}_{2}\;100\;\mathrm{ppb}$	$S=\frac{R_{\mathrm{air}}}{R_{\mathrm{gas}}}$	Room temperature	CO 198 s/36 s ${\mathrm{NO}}_{2}$ 16.03 s/27 s	[44]/ 2022
			$\mathrm{CO}\;0.83/{\mathrm{NO}}_{2}\;1.24$			

## Data Availability

Not applicable.
